# Measurement of diallyl disulfide and allyl methyl sulfide emanating from human skin surface and influence of ingestion of grilled garlic

**DOI:** 10.1038/s41598-019-57258-1

**Published:** 2020-01-16

**Authors:** Shodai Sato, Yoshika Sekine, Yuya Kakumu, Tadahiro Hiramoto

**Affiliations:** 1 Graduate School of Science, Tokai University, 4-1-1 Kitakaname, Hiratsuka, Kanagawa 259-1292 Japan; 2 Innovative Technology Research Laboratory, Takasago International Corporation, 1-4-11 Nishiyawata, Hiratsuka, Kanagawa 254-0073 Japan

**Keywords:** Digestive signs and symptoms, Bioanalytical chemistry

## Abstract

Diallyl disulfide (DADS) and allyl methyl sulfide (AMS) have been known as a metabolic product of sulfur-containing foods, typically garlic. The odour of such organosulfur compounds following garlic ingestion is often considered as an unpleasant element. Although previous studies have identified the DADS and AMS associated with garlic breath, no study has been reported on the determination of both compounds emanating from human skin surface. This study aimed to demonstrate the effect of garlic ingestion on the dermal emissions of DADS and AMS using a passive flux sampler coupled with gas chromatography-mass spectrometry. Firstly, baseline levels were investigated for 30 healthy volunteers in their daily life. The results of 1 h-sampling at the forearm showed the emission fluxes of both compounds followed the lognormal distribution with a geometric mean of 0.18 ng cm^−2^ h^−1^ for DADS and 0.22 ng cm^−2^ h^−1^ for AMS. Subsequently, the garlic ingestion tests were conducted for selected volunteers. The emission flux of DADS increased just after grilled garlic ingestion and decreased gradually thereafter. In contrast, the dermal emission flux of AMS reached a peak at 30 min after ingestion, and then gradually decreased. This peak shift suggests AMS is relatively latent in the skin organs.

## Introduction

Garlic (*Allium sativum*) is a bulb that has been used as a spice, functional food and traditional medicine all around the world since ancient times^1,2^. Ingestion of garlic is known to have many health benefits; it helps in lowering blood pressure and cholesterol^3^ and in reducing the risk of cancer^4–6^. However, Consumption of garlic is empirically known to cause unpleasant breath and body odour which can last from several hours to days^7^. As for breath, previous studies have identified several volatile organosulfur compounds associated with characteristic odour of garlic breath including diallyl disulfide (DADS) and allyl methyl sulfide (AMS)^8–11^. DADS is produced from allicin, a precursor of many organosulfur compounds in the human body and has an odour threshold of 0.22 ppb^12^. It is subsequently metabolized into other compounds including AMS, which has an odor threshold of 0.14 ppb^12^. These compounds were found at a high concentration in human breath immediately after ingestion of garlic^9,10,13^, and AMS was responsible for the persistent garlic odour in breath because digestion of AMS takes several hours. Meanwhile, no study has been reported on the emissions of these volatiles from the human skin surface, even though the ingestion of garlic also causes unpleasant body odour.

Trace gas emanating from the human skin surface is called “human skin gas”. Human skin gas has been attracting considerable attentions in relation to: its role as a mosquito attractant^14^, the specific odour of aged people^15^, indicators for exposure to tobacco smoke and indoor air pollutants^16^, non-invasive medical biomarkers for severe burn status^17^, acute poisoning by agrochemicals^18^, liver disease^19^, and so on. When volatile compounds are formed by the internal metabolism and carried into the blood, they can rise to the skin surface with perspiration and/or travel directly from the blood through the dermal layers, as an expansive network of blood capillaries lies just beneath the skin^20^. Therefore, the release of human skin gas is one of the excretion routes of volatile compounds in the human body. For the determination of emission flux (emission rate per area) of human skin gases, Sekine *et al*.^15–19,21^ developed a simple device based on the concept of a passive flux sampler (PFS). Since PFS uses a molecular diffusion process and does not require any power supply, an investigator can use the sampler anywhere on a non-invasive basis, so volunteers are free from sampling stress. This study aimed to demonstrate the effect of garlic ingestion on the human body odour by determining emission fluxes of DADS and AMS before and after ingestion of grilled garlic using PFS-coupled with gas chromatography-mass spectrometry (GC-MS).

## Results and Discussion

### Method validation

**Figure 1:**
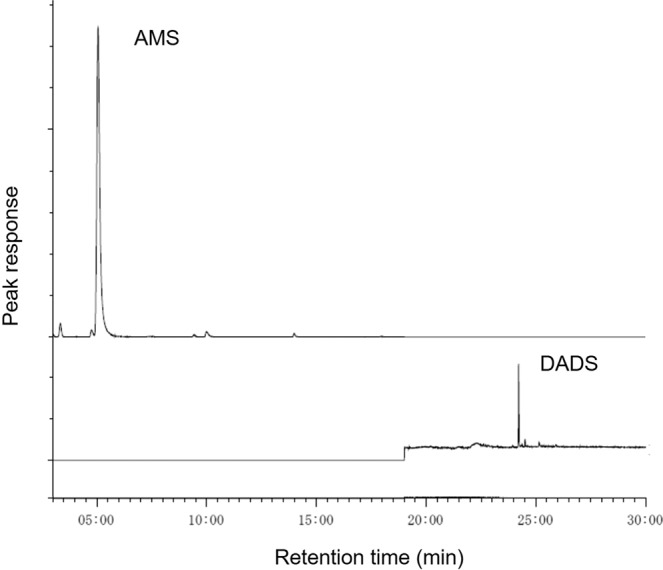
Typical GC-MS chromatograms of AMS and DMDS (10 ng ml^−1^ of mixed standard solution).

Typical GC-MS chromatograms of the mixed standard solution (10 ng mL^−1^ for each component) are shown in Fig. 1. Peaks of both components were well separated with a retention time of 4.1 min for AMS and 24.1 min for DADS. Good linearity was found between the concentration of a dilution series of analytes in methanol (2.0, 5.0, 10 and 20 ng mL^−1^), and their corresponding peak areas: *r* = 0.96 for DADS and *r* = 0.99 for AMS. The recovery rate of the trapped DADS and AMS were investigated by administrating 0.25 ng of both compounds to the DCC trapping medium, which corresponds to an emission of 0.42 ng cm^−2^ h^−1^ for 1 h-sampling. The recovery rates were found to 99.2% for DADS and 98.9% for AMS (*n* = 5). The reproducibility of the passive sampling method was assessed by the simultaneous exposure of five samplers to the vapors of 2.5 ng of DADS and AMS generated from standard solutions. The relative standard deviations of the collected amounts were 1.3% for DADS and 2.8% for AMS (*n* = 5). Since no significant contamination was detected in the blank samples, limit of detection (LOD) was defined as three times the baseline noise level (*S/N* = 3) and resulted in 0.0087 ng cm^−2^ h^−1^ for DADS and 0.0022 ng cm^−2^ h^−1^ for AMS for 1 h-sampling. Based on the excellent recovery rate, reproducibility and sensitivity of our method, we then applied the PFS to the passive sampling of human skin gas.

### Baseline dermal emissions of DADS and AMS (Test 1)

**Figure 2:**
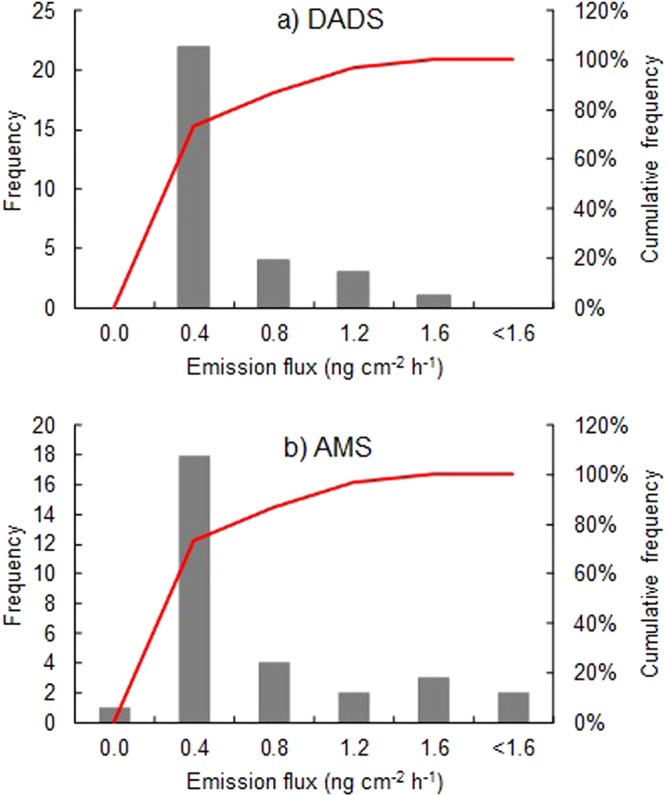
Distributions of emission fluxes of DADS and AMS from skin surface of 30 healthy volunteers, measured by the PFS. (**a**) Histogram and accumulated frequency for DADS (**b**) Those for AMS.

Measurement of dermal emission fluxes of DSDS and AMS were conducted for 30 volunteers to know usual emission levels in their daily life. The volunteers were asked to collect their own human skin gas at the non-dominant forearm by the PFS at any time for 1 h. Figure 2 shows distributions of dermal emission fluxes of DADS and AMS, which are presumed to follow the lognormal distribution (Shapiro-Wilk test, *p* < 0.001 for DADS and *p* < 0.001 for AMS). The emission flux of DADS ranged from 0.011 to 1.6 ng cm^−2^ h^−1^ with a geometric mean of 0.18 ng cm^−2^ h^−1^ (*n* = 30), whilst that of AMS ranged from below LOD to 1.7 ng cm^−2^ h^−1^ with a geometric mean of 0.22 ng cm^−2^ h^−1^ (*n* = 30). Since this is a first report on the determination of dermal emission fluxes of both compounds, the levels were compared with those of other human skin gases; The levels are approximately one hundredth that of acetaldehyde^21^ and one tenth that of acetone^21^. The results showed the dermal emission of DADS and AMS is a daily occurrence probably because of ingestion of sulfur-containing foods. Diacetyl has been known as a middle-aged male odour because its dermal emission flux significantly depends on gender and age^15^. However, the gender and/or age dependences were not found for those of organosulfur compounds concerned, and no remarkable relationship was found between emission fluxes of both compounds.

### Variations of dermal emissions after garlic ingestion (Test 2)

**Figure 3:**
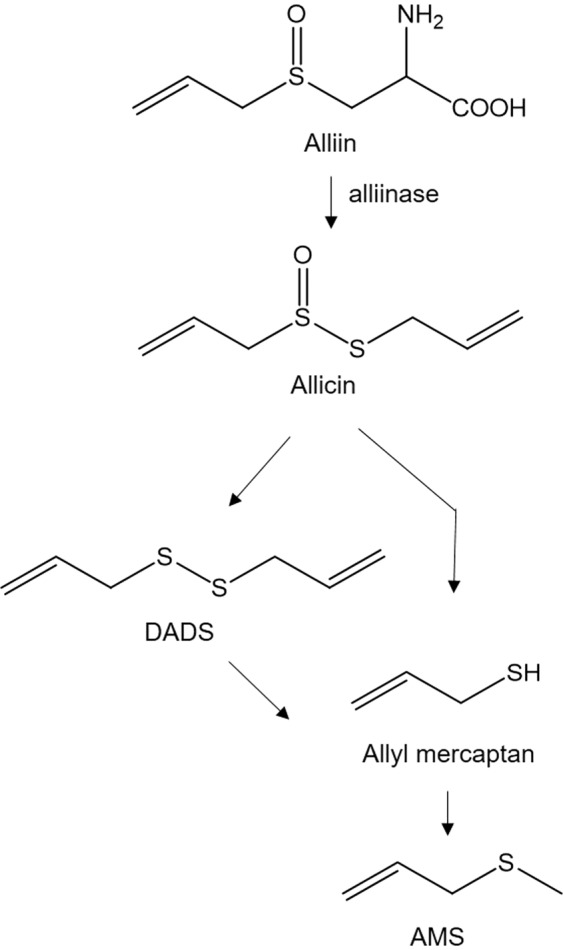
Possible metabolic pathway for the synthesis of AMS from alliin via DADS.

To investigate the effect of garlic ingestion on the dermal emissions of DADS and AMS, variations in the emission fluxes of both compounds before and after ingestion were measured at the non-dominant forearm of three male volunteers A-C. Fresh garlic contains alliin which is a derivative of the amino acid cysteine. Garlic itself has an imperceptible smell, but once fresh garlic is chopped or crushed, alliin is converted to allicin by alliinase (Fig. 3). Allicin is a precursor to many organosulfur compounds that produce the characteristic garlic odour^22,23^. One of the first volatiles formed from allicin in the metabolic pathway is DADS.

**Figure 4:**
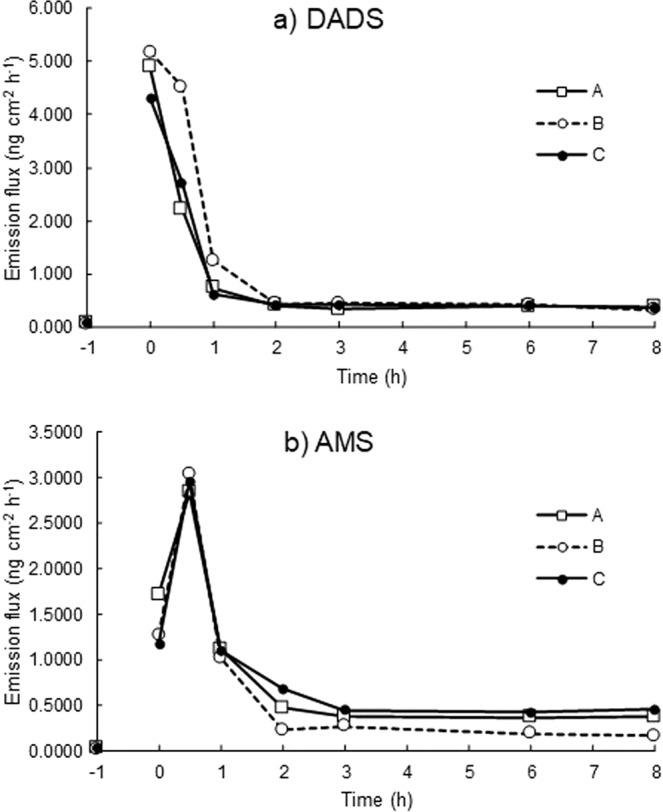
Variations of the dermal emission fluxes of DADS and AMS before and after ingestion of grilled garlic measured at the forearm of three healthy male volunteers A -C (amount of ingested garlic, A: 46 g, B: 45 g, C: 42 g).

**Figure 5:**
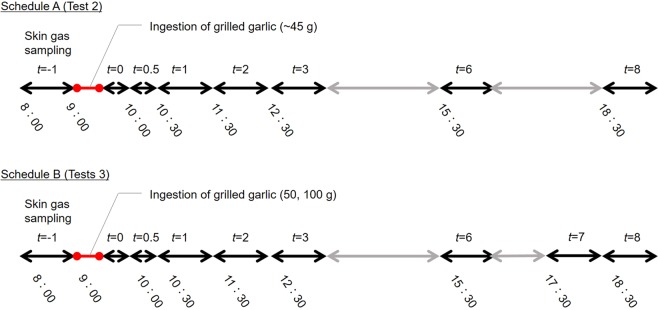
Sampling schedules for the volunteer tests 2 and 3.

Figure 4a shows the time course of the emission flux of DADS from three volunteers before and after ingestion of 45 g of grilled garlic. The time points on the X axis show the start time of samplings after the ingestion (refer to Fig. 5). A common pattern was found for three male volunteers. The initial emission flux of DADS (*t* = −1 h) was observed to be 0.044–0.086 ng cm^−2^ h^−1^ (*n* = 3) at the forearm, which were in the range of daily baseline levels mentioned above. The emission flux increased during the first sampling (*t* = 0 h), reaching a maximum of 4.3–5.2 ng cm^−2^ h^−1^ and then decreased to approx. 0.4 ng cm^−2^ h^−1^ at *t* = 2 h.

In contrast, the time course of the dermal emission flux of AMS differed from that of DADS but was common to all volunteers (Fig. 4b)). The initial emission flux of AMS was 0.020–0.023 ng cm^−2^ h^−1^ (*n* = 3), which are about one third that of DADS and in the range of daily baseline levels. After ingestion of grilled garlic, the emission flux peaked at *t* = 0.5 h with 2.8–3.0 ng cm^−2^ h^−1^, and then decreased to approx. 0.3 ng cm^−2^ h^−1^. The time course differed from that of DADS which peaked at *t* = 0 h, that is, a peak shift was found in the dermal emission flux of AMS.

According to Mirondo^11^, AMS concentration in human breath increased within 5 min after ingestion of garlic. Thus, AMS seems to be formed rapidly through methylation of allyl mercaptan by S-adenosylmethionine and gut microflora^24^ and then exhaled immediately. AMS in breath comes from three possible sources^11^. One possible source is residual food particles containing garlic components in the mouth. The second source is volatiles coming from the stomach. The third route to AMS in breath occurs from the lung via the blood. When AMS is formed from precursors such as allicin, DSDS and allyl mercaptan, it moves into the blood and then into the lungs, where AMS is exhaled out. Meanwhile, dermal AMS after garlic ingestion has only one source: it comes directly from the bloodstream through the dermal layers and is then released from the skin surface in the same way as volatiles formed by the internal metabolism^21,24^ and exogenous chemicals being subjected in the body^15,18^. Thus, our observation on the peak shift suggests that AMS is somewhat latent in the skin organs after being removed from the blood, rather than DADS.

Even though the dermal emission fluxes of DADS and AMS gradually decreased with time, the values at *t* = 8 h, 0.32–0.37 ng cm^−2^ h^−1^ for DADS and 0.17–0.45 ng cm^−2^ h^−1^ for AMS were still greater than the initial values (*t* = −1 h). This means that the body odour caused by DADS and AMS lasted for at least 8 hours after intake of grilled garlic in this case. The dermal emission fluxes of DADS and AMS were compared with the odour thresholds of DADS (0.22 ppb, 1.3 μg m^−3^ at 298 K) and AMS (0. 14 ppb, 0.50 μg m^−3^ at 298 K). A simple calculated ratio of emission flux to odour threshold showed that AMS may contribute as a source of garlic body odour more than DADS, as in a garlic breath.

### Influence of the amount of garlic on the emission flux (Test 3)

**Figure 6:**
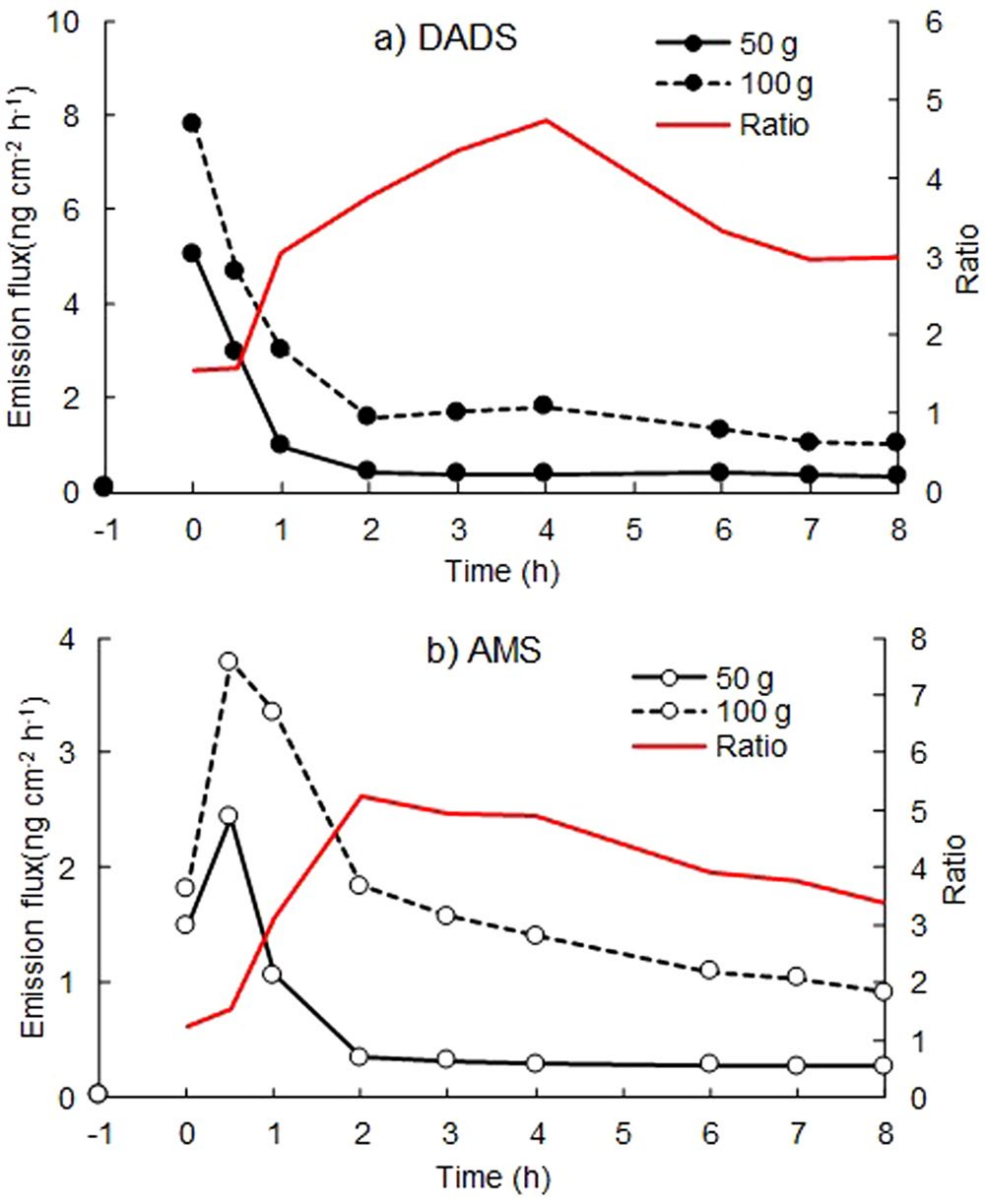
Influence of the amount of garlic on the dermal emission fluxes of DADS and AMS after ingestion of grilled garlic measured for volunteers A and B. Mean emission fluxes are shown. The red line shows ratio of the emission flux at 100 g to that at 50 g of ingested grilled garlic.

The effect of the amount of garlic ingested on the dermal emissions of DADS and AMS were investigated for two volunteers A and B. Figure 6 shows the variations in the dermal emission fluxes of DADS and AMS after ingestion of 50 g and 100 g of grilled garlic. The ratios of the emission fluxes at 100 g and at 50 g were shown together. As was expected, the emission flux increased with an increase in the amount of garlic for both compounds. However, the ratio was not equal to 100/50 = 2 at every sampling time: less than 2 at *t* = 0 and 0.5 h and greater than 2 at *t* = 1 h and after. This is probably because 100 g of grilled garlic was a considerable amount for volunteers to eat within 30 min. So, the digestion of solid garlic pieces might cause a delay in the formation of DADS and AMS and this resulted in less than 2 at *t* = 0 and 0.5 h. Previous studies showed that, for ammonia^19^, acetone^25^ and fenitrothion^18^, the dermal emission flux of human skin gas depends on its blood concentration. Since DADS and AMS are stored temporarily in the body after digestion, the ratio of the emission fluxes became greater than 2 at *t* = 1 h and after in this case.

### Variation of the dermal emission fluxes by sampling positions (Test 4)

**Figure 7:**
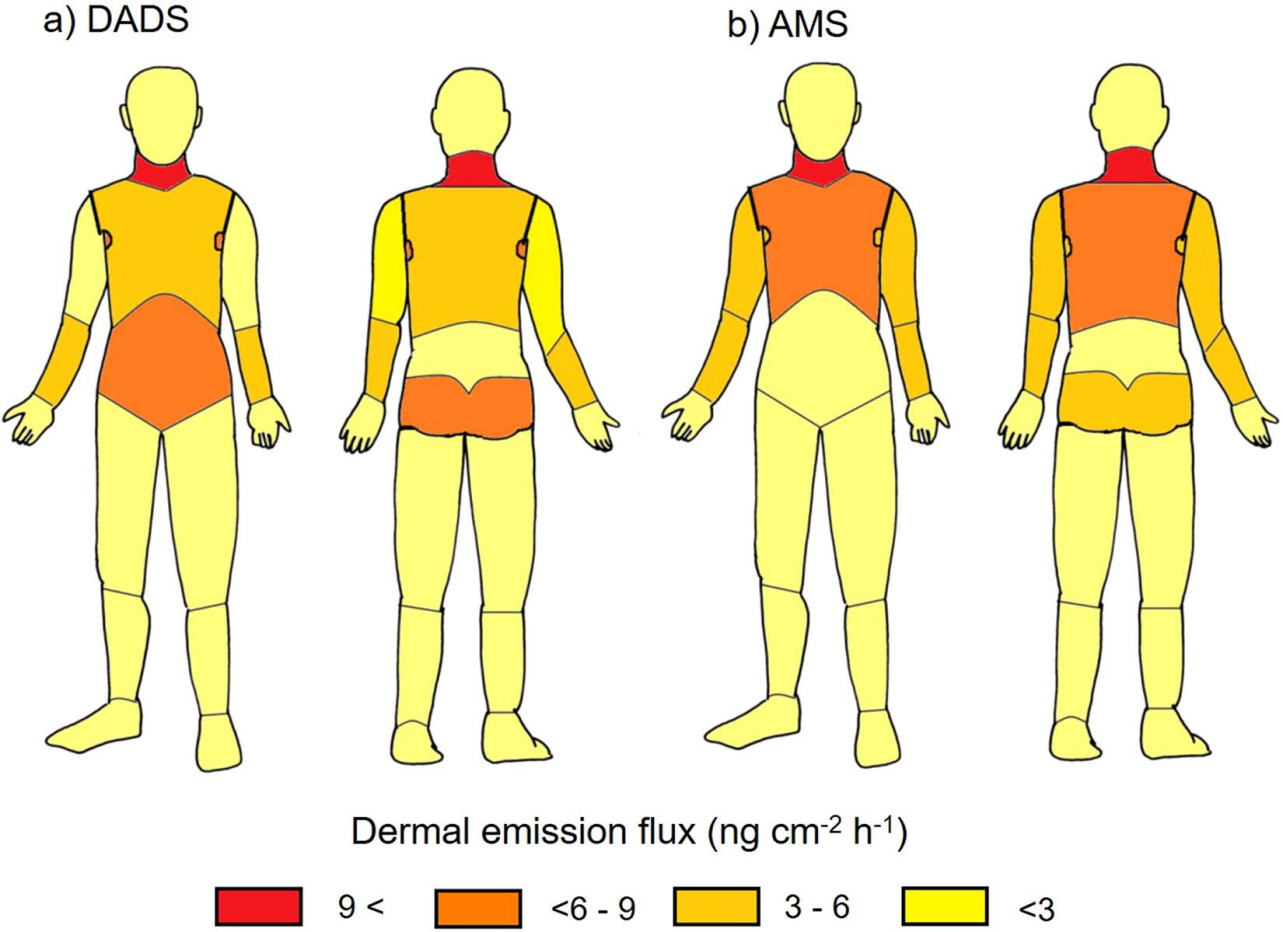
Variation of the dermal emission fluxes of DADS and AMS with the sampling positions after ingestion of grilled garlic (*t* = 0.5 h) measured for volunteer A (amount of ingested garlic, 46 g).

The influence of the sampling site on the dermal emission fluxes of DADS and AMS was investigated by deploying the PFSs simultaneously at 14 positions on volunteer A at just 30 min after ingestion of grilled garlic. Both compounds were detected in all cases, and the emission flux of DADS ranged from 1.9 (foot) to 11 (neck) ng cm^−2^ h^−1^, whilst that of AMS ranged from 1.3 (hand) to 9.3 (neck) ng cm^−2^ h^−1^. Figure 7 illustrates the distribution of the regional emission fluxes. As for DADS, higher emission fluxes were found at the neck, buttock and abdomen, whilst lower fluxes were observed at the foot and hand. Meanwhile, higher emission fluxes of AMS were found in the upper half of the body, including the neck, chest, upper and lower arms, axilla and back.

Chemicals in the blood also move to skin glands such as the eccrine and apocrine glands because the blood plasma is a precursor of sweat. Volatile chemicals are then released as human skin gas. For example, the regional emission flux of acetic acid showed a good linear relationship with the regional sweat rate at rest^26^. However, both DADS and AMS are insoluble in water. So, the higher emissions of both compounds especially at the neck may also be related to sebum secretion with sweat, but explaining this result is difficult at present.

## Conclusion

This is a first report on the determination of DADS and AMS emanating from human skin surface employing the PFS coupled with GC-MS methodology. The baseline emission fluxes of both compounds at the forearm were successfully obtained for 30 healthy volunteers in their daily life probably because of usual ingestion of sulfur-containing foods. Meanwhile, the garlic-ingestion test for 3 selected volunteers demonstrated the emission fluxes remarkably increased after ingestion of grilled garlic, and then gradually decreased toward initial levels. The emission flux of DADS peaked just after garlic ingestion, whilst that of AMS peaked at 30 min after ingestion. This peak shift suggests that AMS is relatively latent in the skin organs after being released from the blood. The emission fluxes of both volatiles changed depending on the amount of garlic consumed. In this study, the emission fluxes of internal garlic metabolites were measured by the PFS-GC/MS methodology. This quantitative determination method of emission flux can be used for further assessment of the contribution of malodorous human skin gas to living environment with a combination to environmental simulation models.

## Methods

### Materials and reagents

Reagent grade DADS, AMS (Fujifilm Wako Pure Chemical, Japan) and methanol (Kanto Chemicals, Japan) were purchased from commercial sources. Garlic was purchased at a local supermarket and was grilled within a piece of aluminium foil. This cookery is a popular way to eat a whole garlic in Japan.

### Passive flux sampler

**Figure 8:**
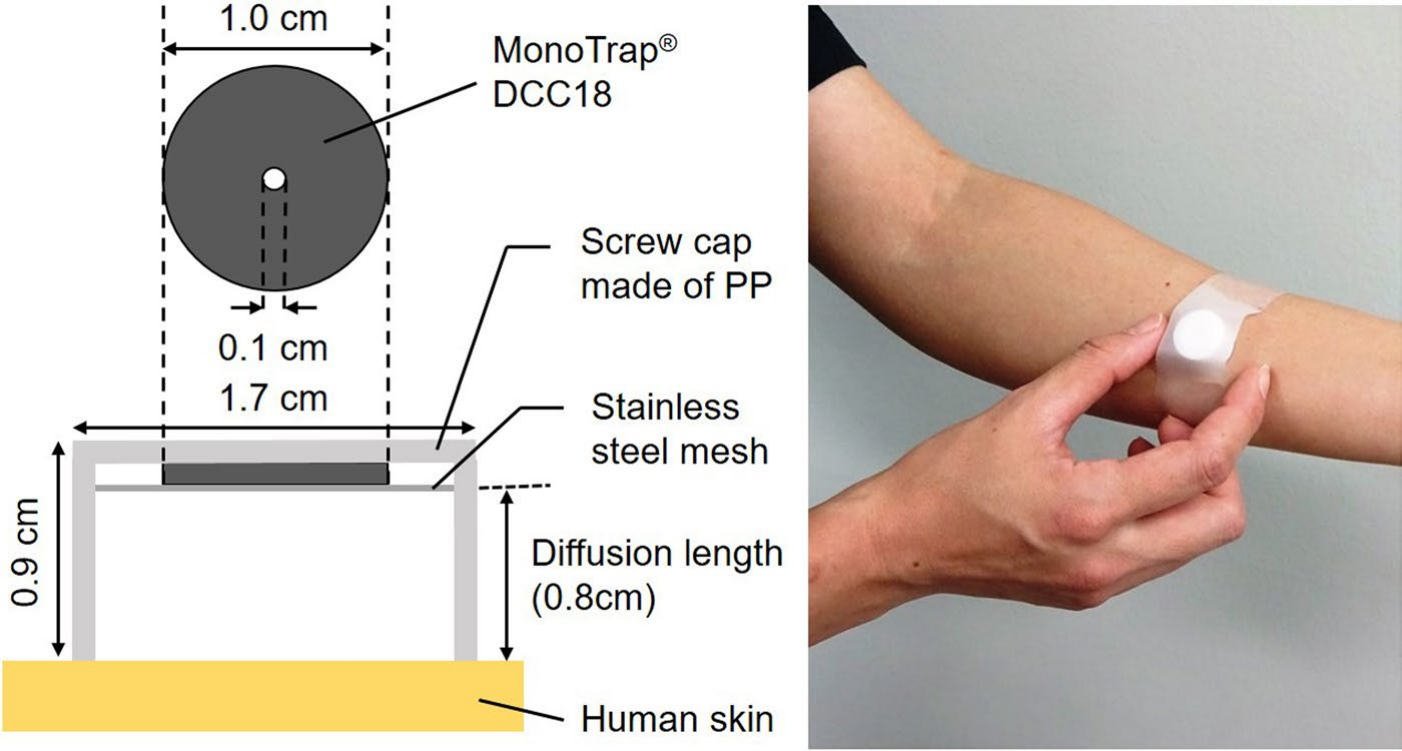
Schematic view of the passive flux sampler for human skin gas measurement and sampling at forearm of a healthy volunteer.

A passive sampling device for collecting human skin gases was developed based on the concept of a PFS as was described in our previous papers^15–19,21^. Briefly, this type of passive sampler was originally developed for the determination of the emission flux of hazardous chemicals from building materials^27^ and was thereafter applied to the skin surface by the authors. The device used in this study (MonoTrap®, SG DCC18, GL Science, Japan) consists of a glass vial with a polypropylene screw cap, a trapping media, and a polytetrafluorothylene (PTFE) O-ring as a stopper. Figure 8 shows a schematic of the PFS when applied to human skin. The PFS is first placed on the skin surface to create a headspace. Through the open face of the sampler, gases such as DADS and AMS emanating from the skin move toward the trapping medium within the headspace by molecular diffusion and the gas molecules are collected on the medium. The diffusion length, which is the distance between the skin surface and the trapping medium in the PFS, was set at 0.80 cm. The DCC18 trapping medium has a large surface area (>150 m^2^/g) provided by its three-dimensional silica monolith network functionalised by hydrophobic octadecylsilyl groups (C_18_H_37_Si) and embedded with activated carbon particles^28^. Prior to use, the open end of the sampler was capped with the glass vial, sealed with a piece of Parafilm^TM^, and enveloped in an aluminium bag.

### Sampling and GC-MS analysis

The PFS was softly fixed on the skin surface of tested healthy volunteers. Before sampling, no special treatment was performed on the skin surface. Sampling duration was 1 h for each sample. The DADS and AMS emanating from the skin surface were collected by the trapping medium and determined using a thermal desorption-GCMS system^16^. After sampling, the trapping medium was transferred into a glass vial and capped. Using a STRAP headspace sampler (JEOL, Japan), the trapped volatile compounds were thermally desorbed at 120 °C and the headspace gases were introduced into a JMS-Q1000GC MkII GCMS system (JEOL, Japan) using a split ratio of 10:1. The volatile compounds were separated using InertCap® Pure-Wax (30 m × 0.25 mm I.D. × 0.25 µm film thickness, GL Sciences, Japan) by employing the following column temperature program: 35 °C hold for 5 min, increase at 5 °C min^−1^ to 100 °C without hold, increase at 25 °C min^−1^ to 250 °C and hold for 6 min. Helium was used as the carrier gas at a flow rate of 1.0 mL min^−1^. Data on the samples were acquired using real time SIM: *m/z* 41 for DADS and *m/z* 73 for AMS. The detector interface temperature was maintained at 250 °C.

Following previous studies^15,18,21^, the emission flux of DADS and AMS, *E* (µg cm^−2^ h^−1^) was obtained by

1 $$E=\frac{W}{S\cdot t}$$

where *W* is the amount (µg) of human skin gas collected by the PFS at a sampling position, *S* is the effective cross-section of the trapping medium (0.594 cm^2^) and *t* is the sampling duration (0.5 or 1.0 h).

Procedure blanks were not considered because no significant peaks of the compounds of interest were found.

### Volunteer tests

The measurement of dermal emission fluxes of DADS and AMS was carried out for healthy volunteers using the PFS to investigate:

- Baseline level of dermal emission fluxes of both compounds in daily life (test 1)
- Variation of the emission flux before and after ingestion of grilled garlic (test 2)
- Influence of the amount of garlic on the emission flux (test 3)
- Variation of the emission flux by sampling position (test 4)

**Test 1**: To investigate usual levels of DADS and AMS emanating from human skin surface of volunteers in their daily life, 30 healthy participants consisting of 18 male (age: 17–59) and 12 female (age: 17–59) volunteers were recruited for the measurement of dermal emission fluxes. The volunteers were asked to collect their own human skin gas at the non-dominant forearm by the PFS at any time for 1 h in their house, school or workplace without any limitation for their act (they were allowed to use their dominant arm during the samplings). No special treatment was conducted for the surface of the forearm before sampling.

**Test 2**: To investigate the effect of garlic ingestion on the dermal emissions of DADS and AMS, variations in the emission fluxes of both compounds before and after ingestion were measured at the non-dominant forearm of three male volunteers A-C (A: age 24, B: age 25, C: age 31) following sampling schedule A shown in Fig. 5. After 1 h-sampling of human skin gas by PFS from 8:00–9:00 (*t* = −1 h), the three volunteers ate ~45 g of the grilled garlic for 30 min using a pair of chopsticks. The amount of garlic ingested was obtained from the weight difference of the dish before and after eating using an electric balance. After ingestion, 0.5 h-samplings were conducted from 9:30 to 10:00 (*t* = 0 h) and from 10:00 to 10:30 (*t*=0.5 h), and then 1 h-samplings were carried out till 18:30.

**Test 3**: To investigate the effect of the amount of garlic ingested on the dermal emissions of DADS and AMS, variations in the emission fluxes of both compounds before and after ingestion of 50 g and 100 g of grilled garlic were measured at the non-dominant forearm of volunteers A and B following sampling schedule B.

**Test 4**: To investigate variations in the dermal emissions of DADS and AMS with respect to sampling positions, the emission fluxes of both compounds were measured for volunteer A at 14 regions. The 14 PFSs were simultaneously deployed on the surface of 14 surface anatomical regions classified by Kurazumi *et al*.^29,30^, namely the head, neck, breast, abdomen, back, lumbar, axilla, upper arms, lower arms (forearm), hands, buttock, thighs, legs and feet. The 0.5 h-samplings were conducted at 30 min after ingestion of 46 g of grilled garlic (*t* = 0.5 h). The volunteer changed his shoes to beach sandals (flip-flops) and remained relaxed and quiet during the sampling process to prevent excess sweating.

The above tests 2–4 were conducted on different days in a study room of the school building of Tokai University Shonan Campus in Kanagawa, Japan. The volunteers A, B and C were forced to refrain from eating garlic and other possible sulfur-containing foods such as leek, onion and so on for 3 days before the tests.

### Ethics approval and consent to participate

This study was performed in accordance with the guidelines laid out in the Declaration of Helsinki and was conducted with the approval of the Institutional Review Board, Shonan Campus, Tokai University, Japan (No. 16181). Written informed consent was obtained from all participants.

## Supplementary information

Figure SI1.

## Data Availability

All data and results have been added to this manuscript.
