# Supplementary material for: Measurement of diallyl disulfide and allyl methyl sulfide emanating from human skin surface and influence of ingestion of grilled garlic
Source: Sci Rep. 2020 Jan 16;10:465. doi: 10.1038/s41598-019-57258-1 (PMC6965658; doi:10.1038/s41598-019-57258-1)
Supplement: Supplementary file 1 — Figure SI1. [file 41598_2019_57258_MOESM1_ESM.docx]

Measurement of diallyl disulfide and allyl methyl sulfide emanating from human skin surface and influence of ingestion of grilled garlic

Shodai Sato, Yoshika Sekine, Yuka Kakumu and Tadahiro Hiramoto

Supplemental Information

Fig.SI 1 Individual dermal emission fluxes of DADS and AMS by age and gender.
